# Magnetic Solid-Phase Extraction of Pyrethroid Pesticides from Environmental Water Samples Using Deep Eutectic Solvent-type Surfactant Modified Magnetic Zeolitic Imidazolate Framework-8

**DOI:** 10.3390/molecules24224038

**Published:** 2019-11-07

**Authors:** Huifang Liu, Lihua Jiang, Meng Lu, Guangyang Liu, Tengfei Li, Xiaomin Xu, Lingyun Li, Huan Lin, Jun Lv, Xiaodong Huang, Donghui Xu

**Affiliations:** 1 School of Life Science and Food Engeneering, Hebei University of Engeneering, Handan 056000, China; 2 Institute of Vegetables and Flowers, Chinese Academy of Agricultural Sciences, Key Laboratory of Vegetables Quality and Safety Control, Laboratory of Quality & Safety Risk Assessment for vegetable Products, Ministry of Agriculture and Rural Affairs of China, Beijing 100081, China

**Keywords:** magnetic solid-phase extraction, zeolitic imidazolate framework, deep eutectic solvents, pyrethroids, environmental water

## Abstract

A simple, sensitive and effective magnetic solid-phase extraction (MSPE) technique was developed for the extraction of pyrethroid pesticides from environmental water samples, followed by gas chromatography tandem triple quadrupole mass spectrometry determination. An adsorbent of magnetic zeolitic imidazolate framework-8@deep eutectic solvent (M-ZIF-8@DES) was prepared using deep eutectic solvent coated on the surface of M-ZIF-8. The features of M-ZIF-8@DES were confirmed by material characterizations, and the results indicated that M-ZIF-8@DES has a good magnetism (61.3 emu g^−1^), a decent surface area (96.83 m^2^ g^−1^) and pore volume (0.292 mL g^−1^). Single factor experiments were carried out to investigate the effect of different conditions on the performance of MSPE. Under the optimal conditions, the developed method performs good linearity (R^2^ ≥ 0.9916) in the concentration range of 1–500 μg L^−1^. The limits of detection were in the range of 0.05–0.21 μg L^−1^ (signal/noise = 3/1). The intraday relative standard deviation (RSD) and interday RSD were less than 9.40%. Finally, the proposed technique was applied for the determination of pyrethroid pesticides in environmental water samples. This work shows the potential of DES-modified metal-organic frameworks for different sample pretreatment techniques.

## 1. Introduction

Pyrethroids are a class of synthetic lipid insecticides which have similar structure to the pyrethrins. In virtue of the high activity, low toxicity and low level of residues in the environment, pyrethroids were regarded as an alternative to the traditional high toxicity pesticides of organochlorine or organophosphorus pesticides in agricultural production [1]. However, the residue of pyrethroids can pollute the environment surroundings and inevitably are harmful to human health or the ecological system because of the excessive application and overtime accumulation of it [2,3]. Therefore, developing an effective and sensitive determination technology for monitoring of pyrethroids in environmental water is imperative. As is well known, sample pretreatment is a key procedure for the analysis of pesticides residue [4]. In recent years, varieties of pretreatment methods, such as solid-phase extraction (SPE), solid-phase microextraction and matrix dispersion SPE, have been developed for the enrichment of pyrethroids in aqueous solutions [5,6,7]. Among them, magnetic SPE (MSPE) is a new type of SPE technique which is based on the magnetic adsorbent, and has gained huge attention in bioseparation and chemical analysis [8]. The typical process of MSPE was as follows: magnetic adsorbents were usually dispersed in the sample solution followed by sonication, shocking or votexing to accomplish the extraction process, and thereafter were retrieved by use of a magnet. Owing to the convenience and high efficiency of magnetic separation process, MSPE can get over the shortage of tedious or time consuming column packing and phase separation of traditional SPE [2]. Recently, some efforts have been made to prepare magnetic adsorbents, such as surface-modified magnetic nanoparticles and magnetic composites composed of magnetic nanoparticles combining carbon nanomaterials, molecularly imprinted polymers or metal‒organic frameworks (MOFs) [9,10].

Zeolitic imidazolate frameworks (ZIFs) is a rising porous polymer, which was constructed by assembling two parts, namely the transition metal ion with imidazole-type organic ligands in a proper solvent [11]. Due to their unique performance of high surface area, excellent chemical stability and abundant structural diversity, ZIFs have drawn dramatic attention in gas storage, catalysis, adsorption and separation of pesticides [12,13,14]. To date, ZIF-67, ZIF-7 and ZIF-8 have been reported in preparing of magnetic sorbents for MSPE purpose. As reported, ZIF-8 exhibits remarkable performance for extraction of chemical pollutants from environmental samples, and it possesses the merits of low-cost and easy preparation [4]. Furthermore, ZIF composites composed of ZIFs and other functional materials such as carbon nanomaterials, ionic liquids or deep eutectic solvents could promote the stability and the adsorption capacity for target analytes of them [15].

Deep eutectic solvents (DESs) are considered to be promising ionic liquids analogues, and were firstly proposed in 2003 [16]. Compared with ionic liquids, DESs are cheaper, easier to prepare, more biodegradable and non-toxic, and herein are in accordance with the requirements of green chemistry [17,18,19]. As a new kind of green solvent, DESs are usually formed by melting two or three cheap and safe components, and appear liquid form ascribe to the hydrogen bonding between hydrogen bond acceptor (HBA) and hydrogen bond donor (HBD). According to literatures, quaternary ammonium salts could be selected as HBAs, and the HBDs are always contain carboxyl and hydroxyl group [20,21,22]. Due to the large number of hydrogen bonding system, DESs have attracted widespread attention in sample pretreatments [23]. In recent years, DESs have been gradually applied to functionalize solid materials such as graphene oxide (GO), carbon nano tubes (CNTs) and magnetic silica due to their unique physicochemical properties and the ease of further modification [24,25,26].

In this study, we aimed to fabricate a composite using DESs modified magnetic ZIF-8 (M-ZIF-8@DES), and applied as an adsorbent for MSPE of pyrethroid pesticides from environmental water samples. After preparation, characterization experiments were operated to investigate its morphology, structure and property. Furthermore, some parameters affecting the extraction performance were optimized through single factor experiments. Finally, the developed analytical method was employed to analyze four pyrethroid pesticides in real environmental water samples.

## 2. Materials and Methods

### 2.1. Materials

#### 2.1.1. Reagents

Liquid standards of the pyrethroid pesticides, including cyhalothrin, cyfluthrin, cypermethrin and flucythrinate, were at a concentration of 1000 mg L^−1^, and were purchased from the Agro-Environmental Protection Institute, Ministry of Agriculture and Rural Affairs of China (Tianjin, China). A 100 mg L^−1^ standard mixture of the four pyrethroid pesticides was prepared in methanol and then stored at −20 °C in the dark. High-performance liquid chromatography grade of ethyl acetate, acetonitrile, methanol, acetone, and *n*-hexane were obtained from Sigma-Aldrich (St. Louis, MO, USA). 1-methyl-3-octyl imidazolium chloride, 1-undecanol and Mercaptoacetic acid were supplied by Beijing Baiweiling Science and Technology Co., Ltd. (Beijing, China).

Analytical grade of ferric chloride hexahydrate (FeCl_3_·6H_2_O), ferrous chloride tetrahydrate (FeCl_2_·6H_2_O), zinc sulfate heptahydrate (ZnSO_4_·7H_2_O), 2-methylimidazole (2-MeIm), and ammonium hydroxide (mass fraction 28%) were acquired from Aladdin Co. (Shanghai, China). Analytical grade of ethanol and all of the other reagents were acquired from the Beijing Chemical Reagents Co. (Beijing, China).

#### 2.1.2. Apparatus

The morphological characteristics of the synthesized materials was measured by Transmission electron microscope (TEM, JEM-200CX, JEOL, Tokyo, Japan) and Scanning electron microscope (SEM, JSM-6300, JEOL, Tokyo, Japan) equipped with energy dispersive spectrometry (EDS). The crystal structure of the compositions was performed according to the X-ray diffractometer (XRD, D8 Advance, Bruker, Karlsruhe, Germany). To determine the magnetic properties of all the materials, vibrating sample magnetometry (VSM, Columbus, USA) was performed with a Lake Shore 7410 magnetometer. The Fourier transform infrared (FT-IR) was carried out using FT-IR-8400 spectroscopy (Shimadzu, Kyoto, Japan). The nitrogen adsorption and desorption isotherms were recorded at 300 K using an ASAP2460 surface area and porosity analyzer (Micromeritics, Norcross, USA). The Brunauer-Emmett-Teller (BET) surface of all the synthesized materials was performed using an ASAP2020 porosimeter (Micromeritics, Norcross, USA).

Gas chromatography-tandem mass spectrometry (GC-MS/MS) analysis was operated by a Shimadzu GC-2010 plus gas chromatograph combined with an AOC-20s auto sampler and a Shimadzu TQ8040 triple-quadrupole mass spectrometer (Shimadzu, Kyoto, Japan). A Rtx-5Ms capillary column (0.25 mm (id) × 30 m, 0.25 μm film thickness, Restek, Belle-fonte, PA, USA) used to separate the pesticides. Helium (99.999% purity) was used as the carrier gas at a constant flow rate of 1 mL min^−1^. The column temperature was initially held at 40 °C for 4 min, then increased to 125 °C at 25 °C min^−1^, ramped to 300 °C at 10 °C min^−1^, and held at 300 °C for 6 min. The total run time was 21 min. The injection volume was 1.0 μL in splitless mode. The specific multiple reaction monitoring (MRM) transitions for the four pyrethroid pesticides are shown in Table 1.

### 2.2. Methods

#### 2.2.1. Synthesis of M-ZIF-8

The M-ZIF-8 composite were synthesized according to a chemical coprecipitation method reported previously with a slight modification [27]. First of all, it is the synthesis of Fe_3_O_4_. FeCl_3_·6H_2_O (1.8 g) and FeCl_2_·6H_2_O (0.8 g) were mixed with ultrapure water (240 mL) and then transferred to a three necked flask, followed by a vigorously mechanical stirring at 80 °C for 30 min. Afterwards, ammonium hydroxide (25%, 10 mL) was added into the solution dropwise, and then the mixture was continuously stirred for another 30 min. Finally, the Fe_3_O_4_ nanoparticles were collected with an external magnet and wash three times with ultrapure water and ethanol by turns.

The synthesis process of M-ZIF-8 was as below: first, 230 µL mercaptoacetic acid was dissolved in 140 mL ethanol. The Fe_3_O_4_ nanoparticles above was dissolved in the mixture solution with stirring mechanically for 1 h at room temperature. Then the mixture was separated by a magnet and washed three times with ultrapure water and ethanol, respectively. Secondly, ZnSO_4_·7H_2_O (0.26 g) was dissolved in 240 mL of ultrapure water/ethanol (1:1, *v*/*v*) and then the obtained black precipitation above was dispersed in the mixed solution with a mechanically stirring for 1.5 h. After that, 20 mL of ethanol contained 0.84 g of 2-MeIm was transferred to the above mixture, and with a continuous stirring for 8 h. Finally, the synthesized materials were separated with an external magnet and washed three times with ultrapure water and ethanol, respectively. The final procedure was dried at 60 °C in a vacuum drying oven for 24 h.

#### 2.2.2. Synthesis of M-ZIF-8@DES

The preparation of DES was followed by previously reported methods with a slight modification [16,17,18]. In brief, 1-methy-3-octyl imidazolium chloride and 1-undecanol were mixed at a molar ratio of 1:2 and keeping magnetic stirring until a homogeneous transparent liquid was formed at 40 °C [28,29].

The M-ZIF-8@DES was synthesized as follows: Firstly, DES (0.53 g) was dissolved with methanol (4 mL) and then the synthesized M-ZIF-8 material (0.8 g) was added into it. After that, the mixture solution was ultrasonicated for 1 h with a replacement of water in every 20 min. Afterwards, the M-ZIF-8@DES composite was collected by an external magnet and washed three times with ethanol. Finally, the M-ZIF-8@DES was dried at 60 °C in a vacuum drying oven for 24 h.

#### 2.2.3. The Process of MSPE

**Figure 1:**
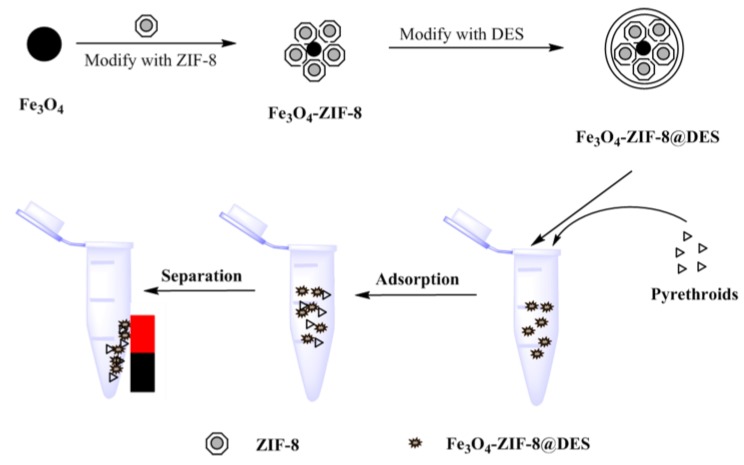
Schematic procedure for the preparation of magnetic zeolitic imidazolate framework-8@deep eutectic solvent (M-ZIF-8@DES) and the steps for the proposed magnetic solid-phase extraction (MSPE) method.

The typical workflow of the MSPE process was shown in Figure 1. Firstly, the adsorbent (8 mg) was added to sample solution (5 mL) in a 10 mL centrifuge tube and shaken for 15 min. Then the mixture was separated by an external magnet, and the supernatant was removed. Subsequently, ethyl acetate (2 mL) was added to the tube with a vortex mixing for 3 min. After desorption, the eluent was separated under external magnetic field, and transferred to another tube. Finally, the eluent was evaporated to dryness with a steady nitrogen flow at 40 °C. The residue was redissolved with acetone (0.5 mL) and a 1.0 µL of this solution was analyzed by GC-MS/MS [30,31].

#### 2.2.4. Preparation of Real Environmental Water Samples

Ground water, well water and river water were selected as real environmental water samples for this study. Among them, the ground water was collected from the Langfang City, Hebei Province, the well water was acquired from the Yanqing District of Beijing and river water was obtained from the Liangshui River in Beijing. All of three samples were filtered through a 0.45 μm polytetrafluoroethylene membrane filter and stored in brown glass bottle at 4 °C.

## 3. Results and Discussion

### 3.1. Characterization of M-ZIF-8@DES

**Figure 2:**
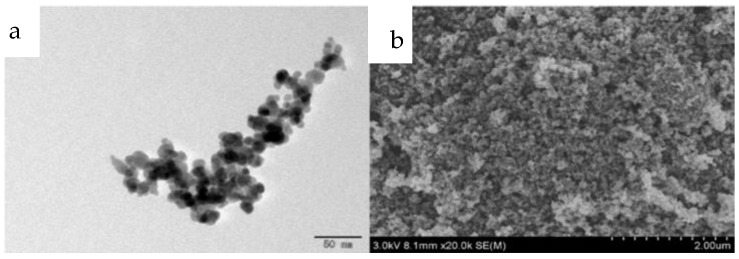
(**a**) Transmission electron microscope (TEM) image and (**b**) Scanning electron microscope (SEM) image of M-ZIF-8@DES.

TEM and SEM were used to examine the morphologies of the prepared materials, and the results are shown in Figure 2. From Figure 2a, we can see that the M-ZIF-8@DES nanoparticles are slightly aggregated. Figure 2b shows that M-ZIF-8@DES possessed a highly dense porous structure, which suggests that the prepared material has good potential in adsorption property.

**Figure 3:**
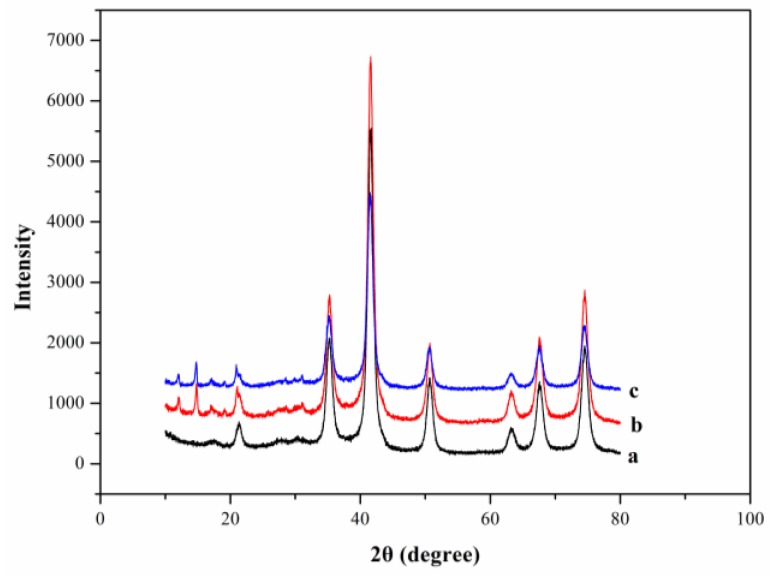
X-ray diffraction patterns of (**a**) Fe_3_O_4_; (**b**) M-ZIF-8 and (**c**) M-ZIF-8@DES.

The crystalline structure of Fe_3_O_4_, M-ZIF-8 and M-ZIF-8@DES were confirmed by XRD. As shown in Figure 3, several characteristic peaks at 21.3°, 35.2°, 41.5°, 63.2°, 67.4° and 74.5° for M-ZIF-8 and M-ZIF-8@DES were in accordance with Fe_3_O_4_. These results suggested that Fe_3_O_4_ nanoparticles were still retained in M-ZIF-8 and M-ZIF-8@DES during the formation of the composite materials [32]. According to the literature, the formation of M-ZIF-8@DES was confirmed by XRD with diffraction peaks in the range of 10–25° [13,18].

**Figure 4:**
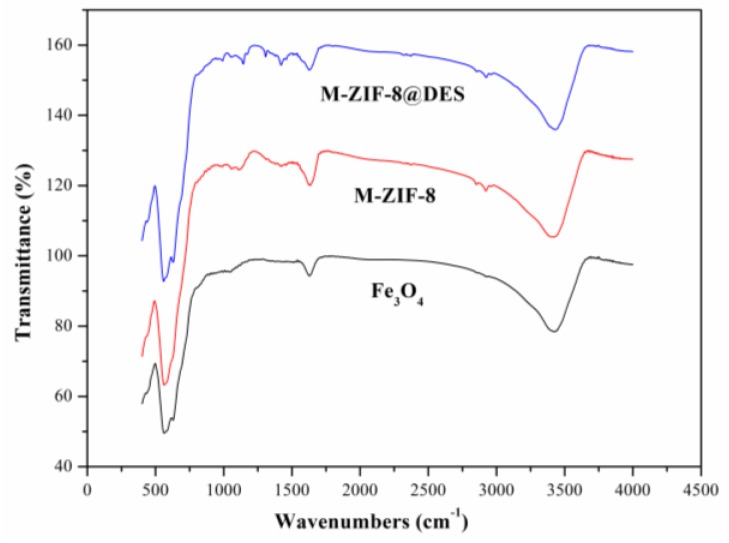
Fourier transform infrared (FT-IR) spectra of the synthetic materials.

The FT-IR spectra of Fe_3_O_4_, M-ZIF-8 and M-ZIF-8@DES are shown in Figure 4. The existence of Fe_3_O_4_ can be confirmed by the characteristic stretching vibration peak of Fe–O at 564 cm^−1^. The adsorption bands at 432, 1418 and 811–1360 cm^−1^ can be attributed to the Zn–N stretching vibration, and the band at 891 cm^−1^ can be corresponded to the stretching vibration of C–N from imidazole ring. Compared to M-ZIF-8, the peak at 1145 cm^−1^ was ascribed to C–N stretch of DES, and the peak at 1456 cm^−1^ was ascribed to the asymmetric –CH3 stretch of DES [33,34]. The above-mentioned results indicated that the Fe_3_O_4_, ZIF-8 and DES were successfully retained in M-ZIF-8@DES composites.

**Figure 5:**
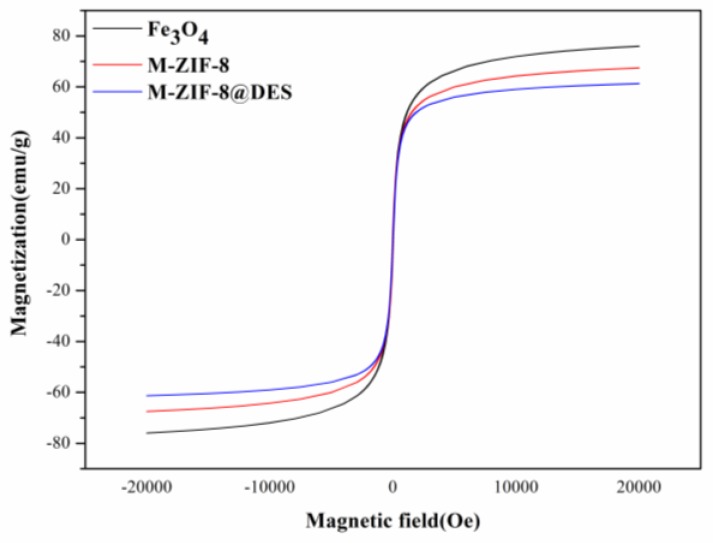
Magnetic curves of (**a**) Fe_3_O_4_; (**b**) M-ZIF-8 and (**c**) M-ZIF-8@DES.

The favorable magnetic properties of the prepared materials are necessary to be applied to magnetic solid-phase extraction. The VSM was used to investigate the obtained materials at ambient temperature. As can be seen from Figure 5, the magnetic hysteresis loops of the three prepared materials show that both the remanence and coercivity values are zero. The results indicated that a typical superparamagnetism feature of the prepared materials and can be rapidly separated with an external magnet. The saturation magnetization values of Fe_3_O_4_, M-ZIF-8 and M-ZIF-8@DES were 75.9, 67.5 and 61.3 emu g^−1^, respectively. All of these results suggest that the M-ZIF-8@DES composites possess good potential as an adsorbent for MSPE.

**Figure 6:**
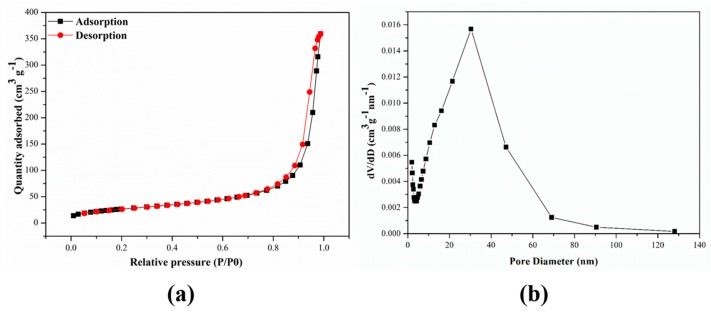
(**a**) N_2_ adsorption and desorption isotherms of M-ZIF-8@DES,(**b**) the distribution of pore sizes of M-ZIF-8@DES.

The N_2_ adsorption-desorption isotherms were used to study the pore properties of the M-ZIF-8@DES and the results were shown in Figure 6a. The shapes of N_2_ adsorption-desorption isotherms were slightly increased at low relative pressures (*P/P0 <* 0.8) and sharply increased at high relative pressure (0.8 *< P/P0 <* 1.0), indicating the coexistence of meso- and macro-pores [4]. The Brunauer-Emmett-Teller (BET) surface area and pore volume of M-ZIF-8@DES were 96.83 m^2^ g^−1^, and 0.292 mL g^−1^, respectively. In addition, the distribution of pore sizes is also presented in Figure 6b. The results indicated that M-ZIF-8@DES has an adequate surface area and total pore volume, which were both favorable for adsorption of pyrethroid pesticides.

### 3.2. Optimization of MSPE Precedure

To identify the optimal conditions of the MSPE procedure, several parameters including the types and amount of adsorbent, adsorption time, types and volume of desorption solvent, desorption time and the pH value of sample solution were investigated by single factor designed experiments [29,35].

#### 3.2.1. Effect of Different Adsorbents

**Figure 7:**
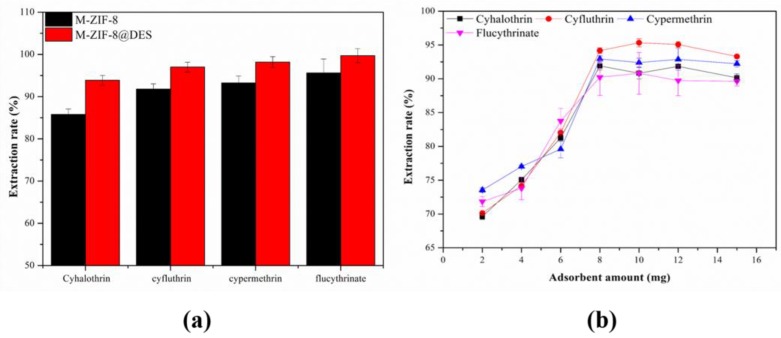
Effect of (**a**) different adsorbents and (**b**) amount of adsorbent on the extraction efficiency.

To evaluate the adsorption capacity of M-ZIF-8 and M-ZIF-8@DES, extraction experiments were performed under the same conditions. From Figure 7a, we can see that both materials possess high adsorption capacity and this might be because of the interactions, including physisorption of ZIF-8 and hydrogen bond formation between target pesticides and adsorbent [36,37,38,39,40]. Furthermore, it is obvious that M-ZIF-8 performed a higher extraction ability when it was modified with DES than M-ZIF-8. This result can be attributed to the probable fresh adsorption sites by coating of DES on the surface of M-ZIF-8. Therefore, coating DES on the surface of M-ZIF-8 based on the “physisorption” mechanism could enhance the extraction ability for the four pyrethoid pesticides [29].

#### 3.2.2. Effect of the Amount of Adsorbent

Considering that the amount of adsorbent has an important impact on the extraction efficiency, 2–15 mg of adsorbent were added to the spiked blank sample solutions (5 mL). As shown in Figure 7b, the extraction efficiencies consistent rise as the mass of adsorbent was ranging from 2 to 8 mg, and then keep relatively constant with the further increasing of the amount of adsorbent. The result demonstrated that 8 mg was the optimal dosage for the MSPE of pyrethroid pesticides and was chosen as the optimal amount of adsorbent for the following experiments.

#### 3.2.3. Effect of the Adsorption Time

**Figure 8:**
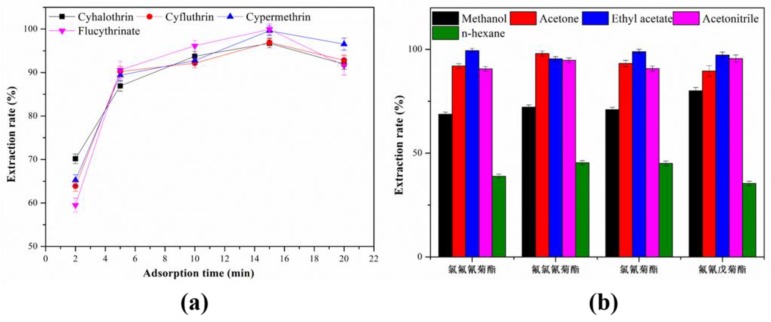
Effect of (**a**) adsorption time and (**b**) different desorption solvent on the extraction efficiency.

The influence of adsorption time on the extraction efficiency was also investigated by the setting of shaking time from 2 to 20 min. As illustrated in Figure 8a, the extraction efficiency increased as the adsorption time increasing from 2 to 15 min, and then kept relatively constant from 15 to 20 min. To take full account of the efficiency of experiment, 15 min was chosen as the optimal adsorption time.

#### 3.2.4. Effect of the Types of Desorption Solvent

The desorption solvent played a vital role in the MSPE procedure. To study the influence of different types of desorption solvent on the desorption efficiency, several organic solvents (methonal, acetate, ethyl acetate, acetonitrile and n-hexane) were selected as potential desorption solvent. As shown in Figure 8b, ethyl acetate gives the best desorption efficiency for MSPE. Thereby, ethyl acetate was selected as the desorption solvent for the following experiments.

#### 3.2.5. Effect of the Volume of Desorption Solvent

**Figure 9:**
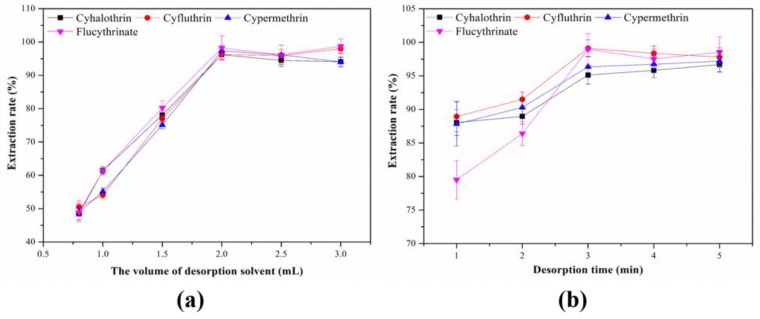
Effect of (**a**) desorption solvent volume and (**b**) desorption time on the extraction efficiency.

In order to investigate the effect of desorption solvent volume on the desorption efficiency, MSPE experiments were carried out with different elution volumes of 0.5 to 3.0 mL. The results of experiment are shown in Figure 9a, the desorption efficiency increased obviously when the desorption solvent volumes ranged from 0.8 to 2.0 mL and then slightly decreased from 2.0 to 3.0 mL. Thus, the optimal desorption solvent volume was set at 2.0 mL.

#### 3.2.6. Effect of the Desorption Time

To study the relationship between desorption time and desorption efficiency, experiments were conducted by setting vortex mixing time from 1–5 min. As shown in Figure 9b, 3 min gives the best desorption efficiency for target analytes. Therefore, 3 min was selected as the desorption time for the following experiments.

#### 3.2.7. Effect of the pH Value of Sample Solution

**Figure 10:**
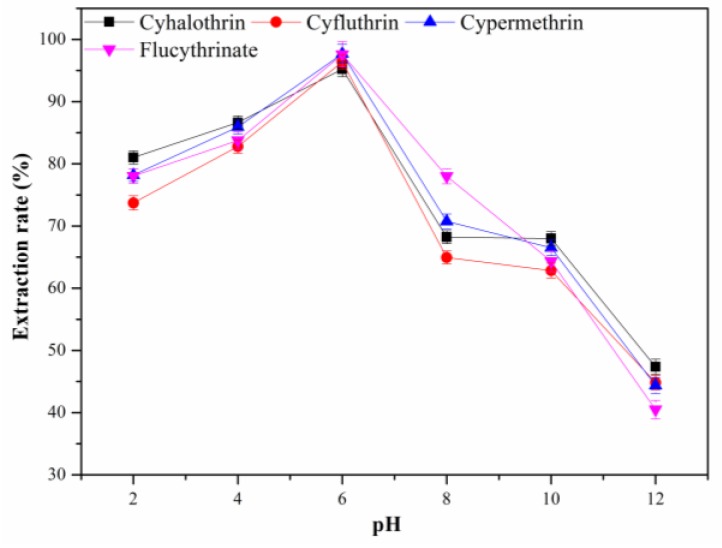
Effect of sample pH on the extraction efficiency.

An appropriate pH of sample solution can probably promote the extraction efficiency for the sample pretreatment. To study the effect of pH on the extraction efficiency of MSPE, the pH value of sample solution was adjusted from 2.0 to 12.0 with NaOH or HCl solution. As shown in Figure 10, the extraction efficiency constantly increased as the pH value increased from 2.0 to 6.0, and then decreased when the pH value increased to 12.0. This could be caused by instability of the pyrethroids under strong alkaline conditions. Hence, the pH value of sample solution was set to 6.0 for the next experiments.

### 3.3. Methodological Study

The validation of the established method was mainly based on the linearity, limit of detection (LOD), and precision by MSPE followed by GC-MS/MS and the results are listed in Table 2. Calibration curves were prepared by analyzing working solutions containing four pyrethiods at concentrations ranging from 1 to 500 μg L^−1^. As can be seen from the table, good linearities were obtained with determination coefficients (R^2^) more than 0.9916. The LOD for the four pyrethroids were calculated with a signal/noise (S/N) ratio of 3, and the result shows that LOD values of the analytes were ranged from 0.05 to 0.21 μg L^−1^. The precision of the proposed method was evaluated by the intra-day and inter-day relative standard deviation (RSD). The results show that RSDs were less than 9.40%. All of these validation results indicate that the proposed method based on MSPE technique has good linearity, low LODs and high precision.

### 3.4. Analysis of the Environmental Water Samples

The developed M-ZIF-8@DES-MSPE-GC-MS/MS-based method was successfully applied to the analysis of four pyrethroid pesticides in three environmental water samples. As shown in Table 3, no pyrethroid pesticides were found in the three real water samples. The recoveries for target analytes from the analysis of spiked real water samples were from 81.1%–97.6%, with the RSDs were in the range of 0.8%–8.2%. Recoveries of the fortified samples indicate that the proposed method can be applied to determination of the four pyrethroids in real water samples.

## 4. Conclusions

In summary, a novel type composite name as magnetic ZIF-8 modified with DES has been synthesized based on the coordination-polymerization and the physisorption mechanism. The prepared M-ZIF-8@DES was used as an adsorbent for MSPE of the four pyrethroids from environment water samples. The results of material characterization indicated that the M-ZIF-8@DES composite possesses high superparamagnetism, a large specific surface area and a porous structure, which can enable the composite adsorption for the pyrethroids. After the optimization of adsorption and desorption parameters, the developed method shows good linearities, low LODs and good precisions. This work provides the basis for development of methods for removal of pesticides from environmental water samples by DES-modified MOFs.

## Figures and Tables

**Table 1:** Acquisition and chromatographic parameters for the four pytethroids.

Pyrethroids	t_R_ (min)	MRM1 (*m/z*)	CE1 (eV)	MRM2(*m/z*)	CE2 (eV)
Cyhalothrin-1	18.785	197.00 > 161.00	8	197.00 > 141.00	12
Cyhalothrin-2	18.962	197.00 > 161.00	8	197.00 > 141.00	12
Cyfluthrin-1	20.304	226.10 > 206.10	14	226.10 > 199.10	6
Cyfluthrin-2	20.398	226.10 > 206.10	14	226.10 > 199.10	6
Cyfluthrin-3	20.461	226.10 > 206.10	14	226.10 > 199.10	6
Cyfluthrin-4	20.501	226.10 > 206.10	14	226.10 > 199.10	6
Cypermethrin-1	20.630	163.10 > 127.10	6	163.10 > 91.00	14
Cypermethrin-2	20.733	163.10 > 127.10	6	163.10 > 91.00	14
Cypermethrin-3	20.793	163.10 > 127.10	6	163.10 > 91.00	14
Cypermethrin-4	20.831	163.10 > 127.10	6	163.10 > 91.00	14
Flucythrinate-1	20.794	199.10 > 157.10	10	199.10 > 107.10	22
Flucythrinate-2	20.985	199.10 > 157.10	10	199.10 > 107.10	22

**Table 2:** Validation of M-ZIF-8@DES as an adsorbent for MSPE of the four pyrethroids.

Pyrethroids	Calibration	Linear Range (μg L^−1^)	R^2^	Intraday RSD (%)	Interday RSD (%)	LOD (μg L^−1^)
cyhalothrin	y = 18526x − 246635	1–500	0.9916	5.52	5.65	0.06
cyfluthrin	y = 16854x − 200703	1–500	0.9946	4.99	5.97	0.21
cypermethrin	y = 38872x − 506601	1–500	0.9940	7.93	9.40	0.17
flucythrinate	y = 56590x − 585207	1–500	0.9934	7.56	7.93	0.05

LOD means Limit of detection.

**Table 3:** Analytical results for determination of pyrethroids in real environmental water samples.

Matrix	Analyte	Spiked Concentration (μg L^−1^, *n* = 3)				
		0	10		100	
		Found	Recovery (%)	RSD (%)	Recovery (%)	RSD (%)
Well water	cyhalothrin	ND	91.6	5.4	96.9	1.5
	cyfluthrin	ND	88.1	4.8	93.9	4.2
	cypermethrin	ND	82.1	8.2	92.7	2.0
	flucythrinate	ND	86.6	3.5	96.0	1.9
River water	cyhalothrin	ND	81.1	2.4	90.8	2.2
	cyfluthrin	ND	90.2	5.2	94.5	2.9
	cypermethrin	ND	84.1	6.3	97.6	3.4
	flucythrinate	ND	85.9	5.5	93.5	3.8
Ground water	cyhalothrin	ND	85.2	6.2	96.5	1.7
	cyfluthrin	ND	82.9	4.3	92.0	1.9
	cypermethrin	ND	86.3	2.5	88.3	0.8
	flucythrinate	ND	82.5	3.7	90.2	4.4
